# Hereditary Pigmented Naevus

**Published:** 1913-01

**Authors:** 


### ABSTRACTS

Hereditary Pigmented Naevus. Dr. Albert Soiland of Los Angeles, *Calif. State Jour. of Med.* Nov., 1912. The cuts, kindly loaned by Dr. Philip Mills Jones, Secretary of the Society and Editor of the Journal, show the result of X-rays, in a month, pushed to the production of a slight dermatitis.

